# Hepatology clinician readiness to provide addiction treatment in hepatology clinics: A mixed-methods formative evaluation

**DOI:** 10.1097/HC9.0000000000000818

**Published:** 2025-10-14

**Authors:** Lamia Y. Haque, Hye Young Choi, Marissa Justen, Yanhong Deng, E. Jennifer Edelman, David A. Fiellin

**Affiliations:** 1 Department of Internal Medicine—Section of Digestive Diseases, Yale School of Medicine, New Haven, Connecticut, USA; 2 Program in Addiction Medicine, Yale School of Medicine, New Haven, Connecticut, USA; 3 Yale School of Medicine, New Haven, Connecticut, USA; 4 Department of Psychiatry and Behavioral Sciences, University of California-San Francisco, San Francisco, California, USA; 5 Yale Center for Analytical Sciences, Yale School of Public Health, New Haven, Connecticut, USA; 6 Department of Internal Medicine—Section of General Internal Medicine, Yale School of Medicine, New Haven, Connecticut, USA; 7 Department of Social and Behavioral Sciences, Yale School of Public Health, New Haven, Connecticut, USA; 8 Department of Health Policy and Management, Yale School of Public Health, New Haven, Connecticut, USA; 9 Department of Emergency Medicine, Yale School of Medicine, New Haven, Connecticut, USA

**Keywords:** addiction treatment, alcohol use disorder, integrated care, liver disease, substance use disorder

## Abstract

**Background:**

Addiction commonly co-occurs with liver disease; however, few patients receive addiction treatment. Although integrated models are emerging, hepatology clinician perspectives on the implementation of alcohol (AUD), opioid (OUD), and tobacco (TUD) use disorder treatment within hepatology clinics are rarely evaluated. We assessed readiness and associated barriers and facilitators to providing treatment of AUD, OUD, and TUD in hepatology clinics.

**Methods:**

A mixed-methods formative evaluation, including a survey and in-depth semi-structured interviews of hepatology clinicians grounded within the Promoting Action on Research Implementation in Health Services implementation science framework, was conducted at a large urban academic medical center from July to September 2022. Practice patterns, attitudes, readiness, barriers, and facilitators toward treating AUD, OUD, and TUD were evaluated.

**Results:**

Among 50 survey respondents (94% response rate; median age 41, 56% female), 35 completed interviews. Overall, 84%, 38%, and 49% routinely asked patients about alcohol, opioid, and tobacco use, respectively. Furthermore, 86%, 52%, and 42% considered it very important to provide AUD, OUD, and TUD treatment within hepatology clinics, respectively. Among prescribers, 41%, 11%, and 34% felt ready to provide medications for AUD, OUD, and TUD, respectively. In addition, 50% preferred hepatology clinicians directly provide AUD treatment, compared with 26% and 38% for OUD and TUD, respectively. Themes emerging from interviews included knowledge gaps regarding addiction treatments, practical concerns such as time constraints, and the need for flexible care models, new clinical tools, multidisciplinary resources, and training to facilitate addiction treatment.

**Conclusions:**

Hepatology clinicians prioritize providing AUD treatment; however, less than half of prescribers feel ready to provide medications. Initiatives to incorporate addiction treatments within hepatology clinics will require tailored approaches that address knowledge gaps and contextual issues.

## INTRODUCTION

Chronic liver disease, the 10th leading cause of death in the United States, can be caused or exacerbated by addiction.^1^ Mortality due to chronic liver disease has been rising, driven by increases in alcohol-associated liver disease (ALD) often stemming from alcohol use disorder (AUD).^2^^,^^3^ HCV infection, another common cause of chronic liver disease transmitted through injection drug use, co-occurs with opioid use disorder (OUD), AUD, or other substance use disorders.^4^^,^^5^ Tobacco use disorder (TUD) can also affect liver health through fibrosis and HCC.^6^^,^^7^

Addiction treatment receipt may improve liver-related outcomes for patients with chronic liver disease. Reducing and abstaining from alcohol improves liver injury across the spectrum of ALD,^8^ and AUD treatment is associated with reduced hepatic decompensation and improved survival.^8^^–^^11^ In patients with HCV and OUD, integrated care leads to high rates of HCV cure and less reinfection among those receiving medications for OUD (MOUD).^12^^–^^15^ Finally, smoking cessation reduces HCC risk, highlighting the need for treating TUD.^16^

Despite the impact of addiction on liver health, few patients with both liver disease and substance use disorders receive evidence-based medication and behavioral addiction treatments.^17^^–^^20^ Patient, clinician, and system-level barriers affect uptake, and this has been described most extensively in patients with ALD.^21^^–^^23^ Although effective medications for AUD (MAUD) exist, over 70% of hepatology clinicians in a national survey never prescribed them, citing barriers such as limited knowledge, time, and resources.^21^^,^^24^ Integrated care models for providing AUD treatment for patients with ALD through multidisciplinary teams are emerging, but are resource-intensive, limiting scalability.^25^^,^^26^ Hepatology clinician–driven medical management approaches^27^ to treat AUD, OUD, and TUD, for which effective medications and behavioral treatments exist and have been delivered in subspecialty medical settings,^28^ may increase access. Although prior studies have examined perceived barriers to AUD treatment among hepatology clinicians, perspectives regarding integrated care models for treating AUD, OUD, and TUD in the context of chronic liver disease, as well as clinician readiness to provide this care, are not known. Therefore, we conducted a mixed-methods formative evaluation to explore practice patterns, attitudes, readiness, preferences, barriers, and facilitators toward treating AUD, OUD, and TUD in hepatology clinics among hepatology clinicians to inform future integration and implementation interventions.

## METHODS

A formative evaluation,^29^ informed by the Promoting Action on Research Implementation in Health Services (PARiHS) implementation science framework, was conducted to assess factors that may impact the implementation of integrated addiction treatment in hepatology clinics.^30^ PARiHS consists of 3 elements, “evidence,” “context,” and “facilitation,” and was chosen as a widely applied determinants framework^31^ used by our team and others for tailoring implementation strategies.^30^^,^^32^^,^^33^ A questionnaire and qualitative interview guide (Supplemental Materials, http://links.lww.com/HC9/C127) informed by prior literature barriers and facilitators of addiction treatment in hepatology care settings^21^^,^^23^ were developed and piloted by members of the study team with expertise in addiction medicine, hepatology, mixed-methods research, and implementation science. The study was conducted in accordance with both the Declarations of Helsinki and Istanbul and approved by the Yale University Institutional Review Board.

### Setting and participants

Hepatology clinicians, defined as attending physicians, gastroenterology/hepatology fellows, nurses, advanced practice providers, and behavioral health providers (psychiatry, psychology, or social work) caring for patients with liver disease, from clinical sites (both general hepatology and liver transplant clinics) affiliated with a single academic center in Connecticut, were invited to participate in the study. Behavioral health providers were affiliated with transplant-specific sites and provided transplant-related care. Clinicians were required to have cared for patients at ≥1 site for ≥6 months to be eligible. A list of all eligible clinicians was compiled from clinic directories, and invitations to participate were emailed. Participants were invited to complete a confidential online survey, followed by a ~30-minute in-depth interview, and were given an information sheet describing the study. Participants provided online and verbal consent as authorized by the Institutional Review Board and were provided gift cards upon study completion. Data collection occurred from July to September 2022.

### Quantitative data

The survey included questions regarding clinician demographics, clinical experience, exposure to addiction medicine training, practice patterns, attitudes, preferences, and readiness to provide treatment for AUD, OUD, and TUD in hepatology clinics. Survey questions were developed to build upon recent literature on hepatology clinician perspectives on treating AUD^21^ by focusing on clinician readiness and preferred models of care for providing treatment for AUD, OUD, and TUD in liver clinics.^21^^,^^32^^,^^34^^,^^35^ Attitudes and readiness to provide treatment for AUD, OUD, and TUD were assessed using a 5-point Likert-type scale.^36^ The survey was administered before completion of qualitative interviews using a sequential explanatory approach in which preliminary quantitative findings informed qualitative data collection.^37^

### Qualitative data

All clinicians who completed the survey were invited to participate in in-depth semi-structured interviews with the aim of interviewing at least the minimum number of participants needed to achieve thematic saturation.^38^ A purposive sampling strategy was used to send an additional round of invitations to potential participants to ensure that the data reflected perspectives from all clinical sites and professional backgrounds.^39^ The interview guide consisted of grand tour questions and probes grounded within the PARiHS framework to elicit perspectives related to the implementation of integrated treatment for AUD, OUD, and TUD in hepatology clinics through the lens of the “evidence,” “context,” and “facilitation” domains, as well as associated barriers and facilitators to complement and build upon quantitative findings. Interviews were conducted via a secure online platform in which they were recorded and transcribed. Interviews were conducted by 1 member of the research team, and transcripts were reviewed for accuracy and de-identified for analysis.

### Data analysis

Demographic variables and clinician characteristics were examined by using medians and ranges for continuous variables and counts and percentages for categorical variables. Responses to questions regarding practice patterns, attitudes, preferences, and readiness to provide treatment for AUD, OUD, and TUD were summarized with frequencies and percentages. Perspectives on readiness and preferred models of care for providing treatment for AUD, OUD and TUD were compared using generalized linear mixed effects models. Qualitative data were analyzed in several stages. A codebook organized by PARiHS domains was developed, reviewed, and refined by the research team. Transcripts were examined line-by-line with codes applied by 2 researchers and subsequently reviewed to adjudicate discrepancies and iteratively refine the codebook. Excerpts organized by code were examined, and themes were generated within PARiHS domains through thematic analysis.^40^ Methodological integration occurred through a building approach in which quantitative findings were used to determine areas of importance that were explored further through in-depth interviews, and data were synthesized using a contiguous approach for integration through interpretation and reporting.^37^ Analyses were performed using SAS 9.4 (SAS Institute) for quantitative data and NVivo software for qualitative data.

## RESULTS

### Quantitative

#### Participant characteristics

Of 53 hepatology clinicians who were invited to participate in the study, 50 completed the survey (94% response rate); among them, 39 had medication prescribing privileges. Participant characteristics are summarized in Table 1. While the majority received some addiction medicine education (100% among behavioral health providers and 72% among nonbehavioral health providers), 26% reported no prior training.

**TABLE 1:** Hepatology clinician characteristics, n=50

Clinician characteristic	Median (range) or count (percentage)
Age	41 (28–86)
Sex	
Male	19 (38)
Female	28 (56)
Prefer not to disclose	3 (6)
Race	
White	28 (56)
Black	2 (4)
Asian	16 (32)
Middle Eastern	1 (2)
Prefer not to disclose	3 (6)
Ethnicity	
Hispanic/Latinx	2 (4)
** ** Non-Hispanic/Latinx	43 (86)
** ** Prefer not to disclose	5 (10)
Clinician type	
Advanced practice provider	5 (10)
Attending hepatologist	20 (40)
Gastroenterology/hepatology fellow	16 (32)
Nurse	5 (10)
Psychiatrist	1 (2)
Psychologist	1 (2)
Social worker	2 (4)
Level of experience	
Years in practice	9.5 (1–60)
Years caring for patients with liver disease	7.5 (1–61)
Number of patients with liver disease seen per week	15.5 (1–55)
Previous addiction medicine training	
Didactic lecture	30 (60)
Outpatient clinical rotation	3 (6)
Inpatient clinical rotation	4 (8)
Continuing education activity	18 (36)
Clinical work experience	2 (4)
None	13 (26)

#### Practice patterns, attitudes, readiness, and preferences

**FIGURE 1:**
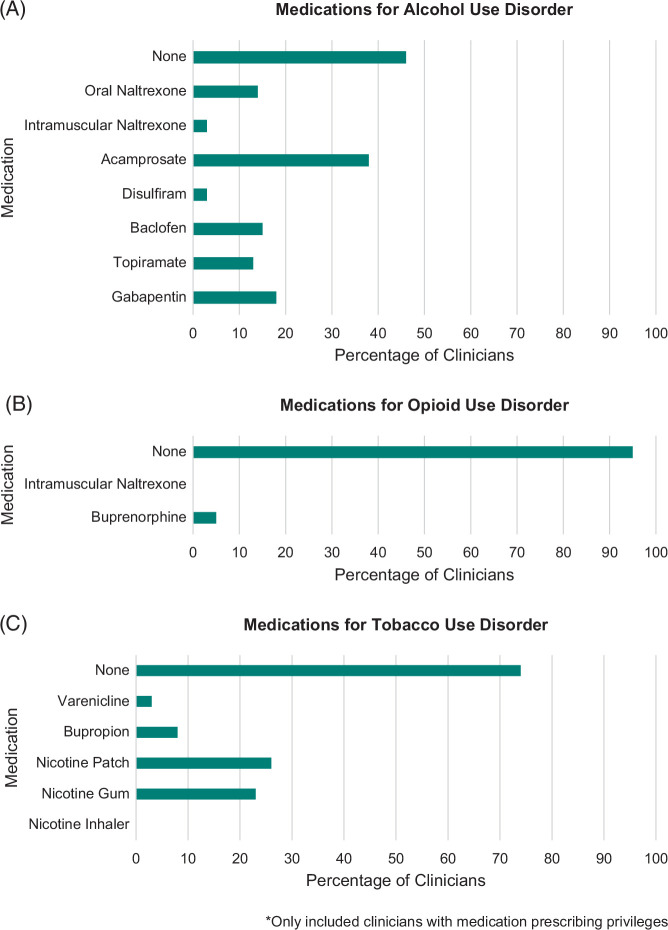
Percentage of clinicians with experience prescribing medications for addiction, n=39*. *Only included clinicians with medication prescribing privileges.

**FIGURE 2:**
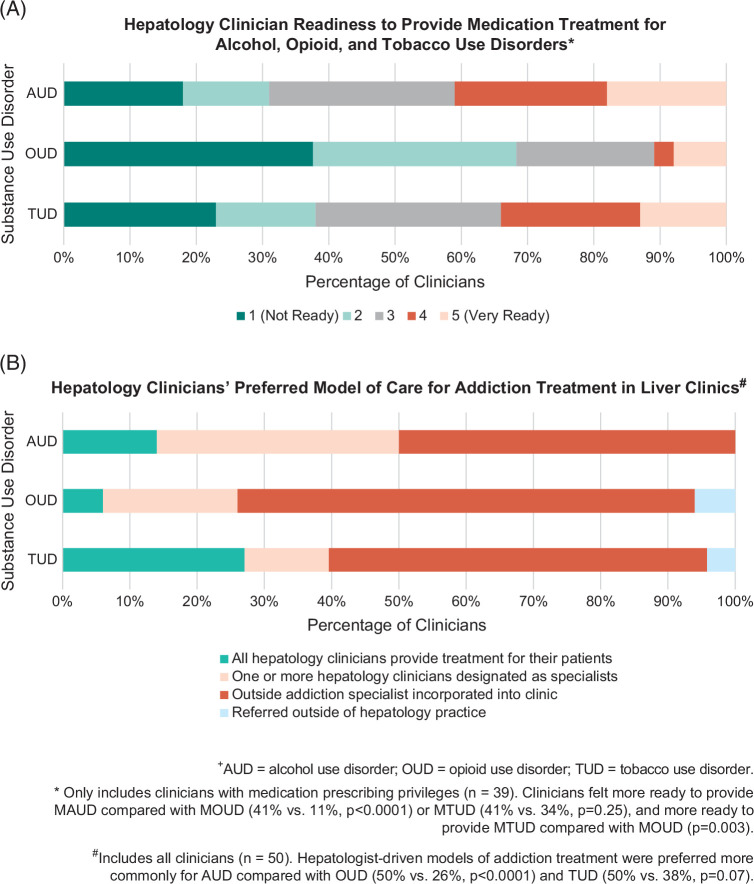
Attitudes and preferences regarding the provision of addiction treatment in hepatology clinics^+^. ^+^AUD, alcohol use disorder; OUD, opioid use disorder; TUD, tobacco use disorder. *Only includes clinicians with medication prescribing privileges (n=39). Clinicians felt more ready to provide MAUD compared with MOUD (41% vs. 11%, *p*<0.0001) or MTUD (41% vs. 34%, *p*=0.25), and more ready to provide MTUD compared with MOUD (*p*=0.003). ^#^Includes all clinicians (n=50). Hepatologist-driven models of addiction treatment were preferred more commonly for AUD compared with OUD (50% vs. 26%, *p*<0.0001) and TUD (50% vs. 38%, *p*=0.07). Abbreviations: MAUD, medications for AUD; MOUD, medications for OUD; MTUD, medications for TUD.

Most reported that at least half of their patients had ALD and AUD, while less than a quarter had OUD or TUD. Alcohol consumption was discussed in >75% of visits by 84% of clinicians (100% among behavioral health providers and 83% among nonbehavioral health providers), whereas opioid and tobacco use were discussed less frequently (both discussed in >75% of visits by 100% of behavioral health providers; opioid and tobacco use discussed in >75% of visits by 33% and 43% of nonbehavioral health providers, respectively). All clinicians referred patients with AUD for addiction treatment, most commonly to specialists in the health care system and community. Referral was less consistent for OUD and TUD, with 10% (0% of behavioral health providers and 11% of nonbehavioral health providers) and 24% (0% of behavioral health providers and 26% of nonbehavioral health providers) of clinicians having never referred patients, respectively. More than half of prescribing clinicians had ever prescribed MAUD, with acamprosate (38%) and oral naltrexone (36%) being the most common (Figure 1A). In contrast, 95% and 74% had never prescribed MOUD and medications for TUD (MTUD), respectively (Figures 1B, C). Practice characteristics and patterns are summarized in Table 2. Most considered MAUD, MOUD, and MTUD to be moderately effective. Provision of AUD, OUD, and TUD treatment in hepatology clinics was felt to be very important by 86%, 52%, and 42% of participants, respectively (Table 3). Clinicians felt more ready to provide MAUD compared with MOUD (41% vs. 11%, *p*<0.0001) or MTUD (41% vs. 34%, *p*=0.25), and more ready to provide MTUD compared with MOUD (*p*=0.003) (Figure 2A). Hepatologist-driven models of addiction treatment were preferred more commonly for AUD compared with OUD (50% vs. 26%, *p*<0.0001) and TUD (50% vs. 38%, *p*=0.07) (Figure 2B).

**TABLE 2:** Practice characteristics and addiction treatment practice patterns among hepatology clinicians, n=50

Practice characteristics and patterns	All clinicianscount (%),^a^ n=50	Behavioral health providers count (%),^a^ n=4	Nonbehavioral health providers count (%),^a^ n=46
Patients seen with alcohol-associated liver disease			
<25%	8 (16)	0 (0)	8 (17)
25%–49%	16 (32)	0 (0)	16 (34)
50%–75%	22 (44)	2 (50)	20 (43)
>75%	4 (8)	2 (50)	2 (4)
Patients seen with hepatitis C infection			
<25%	26 (53)	0 (0)	26 (57)
25%–-49%	19 (39)	2 (50)	17 (37)
50%–75%	4 (8)	2 (50)	2 (4)
>75%	0 (0)	0 (0)	0 (0)
Patients seen with alcohol use disorder			
<25%	10 (21)	0 (0)	10 (22)
25%–49%	13 (27)	0 (0)	13 (28)
50%–75%	21 (44)	2 (50)	19 (41)
>75%	4 (8)	2 (50)	2 (4)
Patients seen with opioid use disorder			
<25%	28 (58)	1 (25)	27 (59)
25%–49%	17 (35)	2 (50)	15 (33)
50%–75%	3 (6)	1 (25)	2 (4)
>75%	0 (0)	0 (0)	0 (0)
Patients seen with tobacco use disorder			
<25%	24 (49)	1 (25)	23 (50)
25%–49%	17 (35)	1 (25)	16 (35)
50%–75%	7 (14)	1 (25)	6 (13)
>75%	1 (2)	1 (25)	0 (0)
Frequency of asking patients about alcohol use			
<25%	2 (4)	0 (0)	2 (4)
25%–49%	1 (2)	0 (0)	1 (2)
50%–75%	5 (10)	0 (0)	5 (11)
>75%	42 (84)	4 (100)	38 (83)
Frequency of asking patients about opioid use			
<25%	13 (26)	0 (0)	13 (50)
25%–49%	10 (20)	0 (0)	10 (22)
50%–75%	8 (16)	0 (0)	8 (17)
>75%	19 (38)	4 (100)	15 (33)
Frequency of asking patients about tobacco use			
<25%	13 (27)	0 (0)	13 (28)
25%–49%	7 (14)	0 (0)	7 (15)
50%–75%	5 (10)	0 (0)	5 (11)
>75%	24 (49)	4 (100)	20 (43)
Frequency of referrals for patients with alcohol use disorder			
<25%	8 (16)	0 (0)	8 (17)
25%–49%	14 (29)	1 (25)	13 (28)
50%–75%	16 (33)	1 (25)	15 (33)
>75%	11 (22)	2 (50)	9 (20)
Frequency of referrals for patients with opioid use disorder			
<25%	24 (49)	0 (0)	24 (52)
25%–49%	12 (24)	2 (50)	10 (22)
50%–75%	7 (14)	2 (50)	5 (11)
**>75%**	6 (12)	0 (0)	6 (13)
Frequency of referrals of patients with tobacco use disorder			
<25%	36 (73)	0 (0)	36 (78)
25%–49%	9 (18)	2 (50)	7 (15)
50%–75%	4 (8)	2 (50)	2 (4)
>75%	0 (0)	0 (0)	0 (0)
Places where patients with alcohol use disorder are referred			
Social worker in the clinic	21 (42)	1 (25)	20 (43)
Psychology or psychiatry services in the clinic	24 (48)	3 (75)	21 (46)
Addiction specialist in health care system	31 (62)	2 (50)	29 (63)
Psychology or psychiatry services in the community	18 (36)	3 (75)	15 (33)
Addiction treatment program in the community	25 (50)	4 (100)	21 (46)
Patients not referred	0 (0)	0 (0)	0 (0)
Other^b^	7 (14)	0 (0)	7 (15)
Places where patients with opioid use disorder are referred			
Social worker in the clinic	19 (38)	1 (25)	18 (39)
Psychology or psychiatry services in the clinic	20 (40)	2 (50)	18 (39)
Addiction specialist in health care system	20 (40)	2 (50)	18 (39)
Psychology or psychiatry services in the community	14 (28)	3 (75)	11 (24)
Addiction treatment program in the community	18 (36)	3 (75)	15 (33)
Patients not referred	5 (10)	2 (50)	3 (7)
Other^c^	5 (10)	0 (0)	5 (11)
Places where patients with tobacco use disorder are referred			
Social worker in the clinic	7 (14)	0 (0)	7 (15)
Psychology or psychiatry services in the clinic	13 (26)	3 (75)	10 (22)
Addiction specialist in health care system	24 (48)	2 (50)	22 (48)
Psychology or psychiatry services in the community	4 (8)	1 (25)	3 (7)
Addiction treatment program in the community	4 (8)	0 (0)	4 (9)
Patients not referred	12 (24)	2 (50)	10 (22)
Other^d^	9 (18)	0 (0)	9 (20)

^a^ If the sum of percentages does not equal 100%, this is due to missing data (<2% of responses per question).

^b^ Includes Alcoholics Anonymous, integrated mental health care, and primary care.

^c^ Includes methadone clinic.

^d^ Includes directly prescribed, 1-800-QUIT-NOW, free programs, smoking cessation clinic, and pulmonology.

**TABLE 3:** Attitudes and preferences regarding provision of addiction treatment in hepatology clinics, n=50

Attitudes and preferences regarding addiction treatment	Count (percentage)
Effectiveness of medication treatment for alcohol use disorder:	
1 (not effective)	1 (2)
2	7 (14)
3	20 (40)
4	13 (26)
5 (very effective)	9 (18)
Effectiveness of medication treatment for opioid use disorder:	
1 (not effective)	1 (2)
2	6 (12)
3	15 (30)
4	12 (24)
5 (very effective)	16 (32)
Effectiveness of medication treatment for tobacco use disorder:	
1 (not effective)	1 (2)
2	14 (28)
3	20 (40)
4	9 (18)
5 (very effective)	6 (12)
Importance of providing alcohol use disorder treatment for patients with liver disease in hepatology clinics:	
1 (not at all important)	0 (0)
2	0 (0)
3	2 (4)
4	5 (10)
5 (very important)	43 (86)
Importance of providing opioid use disorder treatment for patients with liver disease in hepatology clinics:	
1 (not at all important)	0 (0)
2	3 (6)
3	9 (18)
4	12 (24)
5 (very important)	26 (52)
Importance of providing tobacco use disorder treatment for patients with liver disease in hepatology clinics:	
1 (not at all important)	1 (2)
2	5 (10)
3	14 (28)
4	9 (18)
5 (very important)	21 (42)

### Qualitative

#### Participant characteristics

Among those surveyed, 70% were interviewed, including 14 attending hepatologists, 9 fellows, 5 advanced practice providers, 4 nurses, and 3 behavioral health clinicians. Table 4 summarizes key themes that emerged, organized by PARiHS domains, and possible implementation strategies to consider to address barriers and support facilitators of integrated care in hepatology clinics. Regarding fit between qualitative and qualitative results, there was confirmation and expansion of quantitative findings within qualitative results.^37^

**TABLE 4:** Major themes and representative quotes from qualitative interviews describing barriers and facilitators of addiction treatment in liver clinics

PARiHS domain	Key themes (barriers* or facilitators^#^)	Potential implementation strategies	Illustrative quotes
Evidence	Safety concerns, misconceptions, and limited experience regarding medication treatments for addiction among prescribing hepatology clinicians*	Clinician education on pharmacological treatments for addiction	*I think the data for things like naltrexone and baclofen… seem to be fairly promising… I am not averse to risk taking, because all of these drugs can affect the liver, but as long as I’m monitoring the patient, a lot of it has to do with shared decision-making around side effects… it’s also, will this person remain in care, can I make sure they’re not getting too somnolent, etc. It’s the double-edged sword of worrying that you’re going to lose that patient after starting them on a method that needs monitoring because they’re already sick.* *– Attending Hepatologist* *I personally feel that like there’s a lot of fear about using naltrexone with liver disease, and I don’t know all the evidence of how afraid you should be, or if we could take more risks with it, but I also personally feel that acamprosate just doesn’t do a whole lot, and I don’t know all the evidence behind it.* *-Gastroenterology/Hepatology Fellow* *I think what I feel less ready about is how to observe them afterwards, like, how much time do I give it to know what the response is? What should the response be? Do they go to not drinking at all? Or is it just like some percentage reduction that I’m looking for? And when do I switch to a different type of therapy? So I feel like I would be ready to start therapy, but not as much… what to do after that. Are they on this forever?* *– Gastroenterology/Hepatology Fellow*
	Low knowledge of evidence-based strategies to address patient motivation and ambivalence regarding addiction treatment*	Incorporation of motivational interviewing training in educational efforts or adding behavioral counselors with motivational interviewing skills	*Coping with the addiction or dealing with the need for that kind of treatment at the same time as needing paracentesis and lactulose and all the rest of it I think sometimes gets very overwhelming for them.* *– Behavioral Health Clinician* *A lot of the patients aren’t really open to it, and I’m not sure how to change that. I think mostly people are just kind of like, well, I think I know what I have to do, I just need to develop the motivation myself, like no one else can give that to me. And so once I decide myself that I’m going stop drinking, then I’m going to stop drinking. I think I see similar things in people who I try and refer for assistance for weight loss, and it’s just like I need to make that decision and once that’s done, then I don’t really need any help or I already know what they need to do and so I don’t need to hear it from another person.* *– Gastroenterology/Hepatology Fellow* *I think it depends on the patient. I think a few times people have said, ‘Oh yeah, I used to see the substance use disorder people, and that was really helpful, please refer me back.’ But more often, the response I get is like not today, cause they’re not like mentally and emotionally prepared, and then that leaves a gap in starting any medication.* *– Gastroenterology/Hepatology Fellow*
	Desire for more evidence in patients with liver disease to guide the provision of addiction treatment*	Research focusing on treating addiction in patients with liver disease	*If there could be some sort of official trial that like just proved that someone returning to drinking when they have risk of decompensation or cirrhosis is worse than someone with cirrhosis going on naltrexone with this theoretical risk of developing liver failure, and it is actually helping their alcohol use rather than giving them a medication like acamprosate that I feel will not work…* *– Gastroenterology/Hepatology Fellow*
	Challenges in incorporating harm reduction when treating AUD in the context of liver disease*	Clinician training on harm reduction principles	*In opioid treatment, I really do advocate for harm reduction, I remind people if they go out and use, you have to clean needles and like be mindful, because I don’t want to get them reinfected with hepatitis C, but for alcohol I don’t really tell people it’s okay to have a little bit of alcohol, because I don’t see that happening realistically with my patients in a way that’s safe for their liver disease, you know? Which again is two diseases… for their alcohol use disorder they might be able to entertain a drink, but their liver at this point can’t tolerate a drink.* *– Advanced Practice Provider*
	Addiction treatment considered crucial in ALD^#^	Identify and support local champions	*I just had four people die on consults, all under 30, all alcohol. And I was like, I quit, I’m not doing this anymore, because it’s just depressing, right? It’s really heartbreaking, you don’t want to go into this field and feel like you have all these tools, but the most important tool is lacking. It is such a big problem.* *– Attending Hepatologist* *I don’t think that there is a way to overstate how important it is to have addiction treatment and resources available for patients with liver disease.* *– Behavioral Health Clinician*
Context	Limited time and competing priorities in clinic visits affect the ability to provide simultaneous addiction care*	Engage stakeholders to modify clinic visit duration/frequency Design interventions that can be delivered within time constraints Shift addiction treatment tasks to other clinicians	*The patient comes with ten physical complaints, and you have half an hour, and you inevitably run out of time, so I can’t imagine adding another thing that carries a huge emotional load, as well as explaining any drug… so I think that, unless you increase clinic visit times, it would be hard to do.* *– Attending Hepatologist* *There’s not much time for it. To be honest, it is sort of cursory and we provide words of support or encouragement or advice… our focus oftentimes in liver clinic is on medical problems… ascites or volume management or ensuring that their heart rate is at goal on a non-selective beta blocker, and addressing their actual alcohol use problem is not the main focus, so this will sort of get shunted over to another care provider.* *– Gastroenterology/Hepatology Fellow*
	Shortages of addiction specialists and treatment programs limit access for patients*	Expand addiction treatment workforce and capacity among existing clinicians Co-locate addiction treatment services	*There aren’t a lot of people… I think there’ll be a few centers that have this one very specialized person who’s able to do that.* *– Attending Hepatologist* *I think in the current climate… mental health clinics are just really full… and it’s actually really hard to get people into treatment, it requires a lot of work, either for the social worker or the patient themselves just trying to get a call back trying to get the intake… especially if they’re sick or working… so having someone available as part of the team, I think would be really good.* *– Behavioral Health Clinician*
	Social determinants of health and other stressors may impact patients’ ability to access and benefit from addiction treatment*	Collaboration with resources to support patients in need	*Add on the barrier of transportation and cost, and some of these things are not covered by insurance.* *– Attending Hepatologist* *There’s some stuff that you can’t do anything about… they can’t pay the rent and they’re going to be kicked out of their home - like these are stressors that you can’t really manage… they can be just a few steps away from becoming homeless and that’s not something you can fix.* *– Gastroenterology/Hepatology Fellow*
	Stigma at multiple levels hinders the ability to connect patients to addiction treatment*	Interventions to promote patient-centered approaches to care, including education and the use of medically appropriate and person-first language	*Patients are sometimes resistant… there’s a real sense of I think shame a lot of times… and sort of bewilderment that it’s gotten to that point, and they don’t want to think that they need any kind of treatment.* *– Behavioral Health Clinician* *When you made use of specific terms like ‘we’re not here to judge you, we understand that this is a disease that needs to be treated, we want you to get better, we want to help you with this,’ that sometimes helps.* *– Registered Nurse*
	Patient preferences regarding care models for addiction treatment delivery are not known*	Develop patient-centered and patient-informed approaches to delivering addiction treatment	*Understanding what the patients want… what are they looking for? Do they want their physician to address their alcohol use? We want to address their alcohol use, but are we the right people to do so?* *– Gastroenterology/Hepatology Fellow* *I think things that I’ve heard were something like, ‘I don’t want to trade one addiction for another, I’d rather not be dependent on anything, or I know someone who tried this, but they still did drugs anyways, so what’s the point,’ so I think it’s also still hard for some people to accept medications. But I think there are still some people who are interested in it.* *– Gastroenterology/Hepatology Fellow*
	Limitations in reimbursement and financial support*	Alternative payment models with a focus on population health	*I think the hard part is just, and maybe I’m jaded, is everything in this world seems to come down to money and sort of like justifying the benefits of including team members and treating this disease, like this certainly doesn’t bring in more money.* *– Attending Hepatologist* *I think a lot of people just think about that initial investment in building a program like this, because it’s expensive and you really have to tailor it to each individual, that there’s no one size fits all, and so to be able to do that, I think a lot of people are probably nervous about that initial cost.* *– Gastroenterology/Hepatology Fellow*
	Fellows need the guidance of supervising attending physicians*	Education and training for all members of the clinical team	*The prescription just doesn’t get given in that moment - whether the patient wants more time to think about it, or being a fellow, if the attending doesn’t feel comfortable prescribing it and wants the referral to be to addiction medicine or psychiatry.* *– Gastroenterology/Hepatology Fellow* *If the people supervising me were like, hey, you haven’t said anything about what you want to do about their alcohol use… because ultimately, I become comfortable with what my attendings are comfortable with, right?* *– Gastroenterology/Hepatology Fellow* *Sometimes I feel frustrated at my own inability to feel comfortable initiating treatment. It is something that I wish I could do.* *– Gastroenterology/Hepatology Fellow*
	Alcohol use routinely discussed during clinic visits^#^	Provide clinician audit and feedback on the facilitation of treatment	*We definitely always address the alcohol use. Sometimes it feels like we do outsource the management.* *– Gastroenterology/Hepatology Fellow* *We often have the addiction medicine fellows here in the liver clinic but then it’s another person they have to meet, and what I hear often from patients is, ‘Oh, yeah, that would be fine but maybe not today,’ because… a referral to an addiction medicine doctor is another conversation as opposed to like me just addressing it myself, because we’ve already talked about it, and they’re engaged on the subject.* *– Gastroenterology/Hepatology Fellow*
	Opioid and tobacco use not prioritized compared with alcohol and other liver-related concerns*	Clinician education regarding the liver-related impact of OUD and TUD, as well as the TUD impact on morbidity and mortality	*From what I see mostly in clinic, definitely our biggest need would be alcohol. And then you know, probably tobacco. I mean I don’t see that many, or at least I haven’t come across that many people with opioid addiction.* *– Advanced Practice Provider* *I feel like we’re very focused on the alcohol, and maybe not so much on the opioid, and then certainly tobacco, unless you’re in transplant [evaluation], I feel like that goes out the window… all these people are so ill, you know, we’re caught up in the critical issues and we’re very focused on the alcohol.* *– Advanced Practice Provider* *I think I would just need to be convinced more as a liver provider, why it’s so relevant for us to take this on in clinic… I understand the alcohol part, and I understand referencing those other things, but for us to have evidence-based interventions specifically for tobacco and opioid use, I’d have to really understand why it’s so central to their liver care that it would require interventions that are within our clinic setting, and not within other clinic settings… It’s not that I don’t see any relevance to it, I just wonder like if it becomes a distraction from all the other things that we need to take care of for their liver care.* *– Gastroenterology/Hepatology Fellow* *If it does come up, it often is brief and very much sort of ‘you should talk to primary care.* *– Gastroenterology/Hepatology Fellow* *We don’t usually ask about tobacco and opioids, you know, especially if their numbers are good, usually we don’t.* *– Registered Nurse*
	Liver clinic is a setting in which addressing addiction treatment can be relevant, acceptable, and impactful^#^	Integrated, co-located, hepatology clinician–driven interventions for addiction treatment	*Patients feel that they are being sent in many different directions in general, but especially in the context of liver disease and addiction, my sense is that patients would really value a stronger involvement of their liver clinicians in the management of their addiction… I think that there’s probably a stronger bond with the patient, they’re involved in these very important aspects of patients’ care… many patients are concerned about the liver safety of medications because they’re afraid that these could potentially harm their liver, and having the oversight of their liver doctor basically affirming that this is something that they can do and should do I think can be a really powerful.* *– Attending Hepatologist* *I think the best system is a patient-centered system so meeting the patients, where they are and having somebody integrated in the liver clinic and at the same visit they get their liver care and also their substance use disorder care, just to keep it in the environment they are used to, to me that’s much easier to be accepted by patients.* *– Attending Hepatologist* *When somebody has had a long period of illness and has been on the waiting list forever and experienced anxiety waiting for a transplant, having to go for paracenteses frequently or having episodes of encephalopathy and just being hospitalized time after time, their motivation to avoid that set of circumstances is very powerful…they are much more likely to want to avoid alcohol.* *– Behavioral Health Clinician* *If we couched it in the medical therapy part of it, that might be more acceptable if they’re perceiving themselves as being in a medical visit.* *– Gastroenterology/Hepatology Fellow*
Facilitation	Flexible models of care are needed depending on clinician preferences and context^#^	Engage hepatology clinicians in designing models of care that fit their needs while incorporating multidisciplinary and stepped care approaches	*I don’t prescribe those medications but I would be certainly willing to consider that and go through training because I definitely think… as much as we’re ordering paracentesis and albumin and prescribing diuretics and beta blockers, this is something that we can also prescribe; I don’t feel confident or comfortable at this point but I think that anybody who’s involved with those populations would benefit from training in that area.* *– Advanced Practice Provider* *There is a sense by many patients of being judged, there still is substantial stigma… I think that to the degree to which active engagement of [hepatology] clinicians in this process of treating addiction that can help affirm that we as clinicians are not passing judgment at all, but rather really trying to partner in their care and that we that we address this as a medical disease, this is something that can really be very hopefully reassuring and affirming for patients who face these issues.* *– Attending Hepatologist* *It is something feasible to be done in hepatology clinics… to be comfortable in treating the easy patients and have patterns of referral for the more complicated patients.* *– Attending Hepatologist* *I think it has to be a multi-disciplinary approach. I think everybody wishes there was a magic pill, but there really isn’t, and I think there has to be a medicine component and then there has to be like a one-on-one therapy component.* *– Attending Hepatologist* *Whether that’s in incorporated directly within liver practices within partnerships with other specialists who have that expertise as a collaborative program or some type of mentoring program where hepatologists are mentored by addictions medicine specialists, those are things that, probably there’s no one right way to do it, but a combination of those could work.* *– Attending Hepatologist* *With complete respect for all the professions that you mentioned, addiction treatment is probably best managed by an addiction treatment specialist, somebody whose focus is addictions, and not a behavioral health psychologist who does some addiction work and not a psychiatrist who does some addiction work but somebody whose main focus of work is addictions… Optimally I would think that the addictions folks would be housed within the clinic so that it is perceived as seamless by patients.* *– Behavioral Health Provider* *I think we still have to decide like, are we the ones to take this on, or do we integrate it into a multidisciplinary setting but have another member of the team be the main person, and it’s not that I want to give up on it as something that I do as a doctor, it is more just understanding what makes the most sense to make this effective and actually sustainable for the long run.* *– Gastroenterology/Hepatology Fellow* *I also don’t know that it’s serving the patient for it to be coming primarily from a liver doctor. I think it’d be very comfortable as the prescriber, but I would feel burdened if I was also feeling like I was taking over the mental health component of addiction, and that I would rather be kind of supporting another provider that’s embedded in a clinic, or embedded in a multi-disciplinary group than being the person that was primarily performing that service.* *– Gastroenterology/Hepatology Fellow* *Another statistic that I like to sort of point to when we’re thinking about this is, you know, the largest group of doctors that treats depression and anxiety in the United States is not psychiatrists, but primary care physicians, because of the large number of people who have those conditions and just the lack of access to psychiatric care. And when I talk to psychiatrists about this, you know, they feel that primary care doctors are completely as qualified as they are to treat these conditions, and they’re really the ones that are meant to treat the more severe and, you know, harder to manage psychiatric conditions, so I think that same analogy could be used for you know, opioid use disorder and alcohol use disorder. If it is just that on its own, I think we should feel well qualified to treat it, but if there are other comorbidities, that’s where I’d like that extra help.* *– Gastroenterology/Hepatology Fellow*
	Shifts in professional culture can bring AUD treatment into the purview of hepatology care^#^	Engagement of professional societies and credentialing bodies to incorporate AUD treatment into standard training and practices	*We need to… change the culture, so that everybody would be expected to know that.* *– Attending Hepatologist* *We as a field need to decide whether or not we would have an active hand in trying to reduce the risk of recidivism - not just identify the risk of recidivism when it comes to decisions for transplant.* *– Attending Hepatologist* *Only a very small subset of transplant or non-transplant hepatologists have established addiction pharmacotherapy within their clinical practices, and that may represent a cultural shift, a paradigm shift. That will take some time where hepatologists will be able to be formally trained and become comfortable with how these are responsibly administered.* *– Attending Hepatologist* *Some people might be thinking, well we have addiction medicine, we should use it, they’re the experts in this area, of course we should send them over to them, they’re going to do a better job than we would, but, you know, maybe that’s not the case, or the very severe cases can be sent, but there’s probably stuff that could be managed by a hepatologist so… one: education for better understanding, and two: change the culture.* *– Gastroenterology/Hepatology Fellow* *I definitely think that the hepatologist should be able to have comfort with and be able to prescribe these medications. I mean when we see patients with hepatitis C it’s not like we defer to infectious disease to treat the virus… we manage HCC [hepatocellular carcinoma], we don’t always defer patients to oncology… when a patient has fatty liver disease, we address it directly with diet and exercise intervention… And so I don’t see why we would isolate this other than the fact that it’s not comfortable, and it’s not what we’re doing. So I think it should certainly be within scope, especially because it’s not like it’s an esoteric cause of liver disease, it’s a really common cause of liver disease, we’re going to see it all the time.* *– Gastroenterology/Hepatology Fellow*
	System-level investment of resources can support multidisciplinary teams^#^	Engagement of stakeholders, including clinicians and patients, to identify and invest in specific needs	*Comprehensive care - it requires money, time, and people.* *– Attending Hepatologist* *I think the part that is going to be more difficult… is trying to create a clinical environment that welcomes these patients and then kind of relieves the burden of seeking treatment off of them.* *– Attending Hepatologist*
	New clinical tools can be developed and implemented to facilitate addiction treatment^#^	Development of clinical decision support tools and standard procedures for screening, treatment, and referral	*Something very basic, like an algorithm - like if you see a patient with good kidney function, or if they have good liver function, then do this or that.* *– Advanced Practice Provider* *There is no present standardization of that evaluation process right now… something that can be implemented in the future.* *– Attending Hepatologist* *I think it would be really helpful for me if there was a clear sheet that had the list of available medications, with here’s when you use this.* *– Gastroenterology/Hepatology Fellow*
	Awareness of local addiction treatment options and ways to refer can be improved^#^	Increasing familiarity and collaboration with community addiction treatment programs	*Educating them [hepatology clinicians] about what resources are available and how to access those resources in a way that everybody can use it, and it’s not just this black box of a random referral… I think there are a lot of barriers from the physician side, there’s a lack of education.* *– Attending Hepatologist*
	Clinician education and training can be provided at all levels^#^	Implementation of educational and training interventions with clinician input, such as brief lectures, self-directed learning, academic detailing, and mentorship	*There’s education… sitting down with the providers… and educating them about the medications available so they feel comfortable in prescribing them.* *– Attending Hepatologist* *And it may even be like a mentoring thing, with an addiction provider looking at your chart and saying… that makes sense; maybe they’re not treating the patient, but they might give you tweaks.* *– Attending Hepatologist* *I think we should be able to provide basic addiction treatments and recognize or have somebody else help to point out which patients really need more specialized care…I think it all starts with education.* *– Attending Hepatologist* *I’m sure there’s going to be certain people who are interested in this who would be more than willing to get extra training in all of it, but I think the reality is that we all see these patients, and there’s no way to just siphon them into one clinic. The reality is just that everyone sees this in some form or another and will benefit. So I guess, like the earlier and earlier it is in medical training, probably the better.* *– Gastroenterology/Hepatology Fellow*

"*" indicates themes that are considered barriers.

"#" indicates themes that are facilitator.

Abbreviations: ALD, alcohol-associated liver disease; AUD, alcohol use order; PARiHS, Promoting Action on Research Implementation in Health Services.

#### Themes within the evidence domain

Overall, clinicians reported having limited experience providing addiction treatments. Several highlighted concerns regarding the safety of MAUD in patients with liver disease, as well as uncertainty in evaluating the effectiveness of AUD care. Some felt that more evidence on the use of MAUD among patients with liver disease was needed. Aside from behavioral health providers, most clinicians were unfamiliar with behavioral treatments. Many identified anticipated patient ambivalence and lack of comfort with harm reduction strategies as challenges. Although knowledge gaps existed, all clinicians were familiar with the impact of AUD on liver health and felt that AUD treatment for patients with ALD was critical.

#### Themes within the context domain

Multiple themes related to contextual factors emerged. All clinicians discussed alcohol consumption with their patients, given its direct impact on the development and progression of liver disease and the abundant evidence demonstrating the benefits of abstaining from alcohol on liver-related outcomes. Clinicians were less consistent in discussing opioid and tobacco use as they felt they were less directly relevant to liver disease and, therefore, less of a priority than alcohol and other liver-related issues. Clinicians placed greater emphasis on facilitating treatment for OUD and TUD among patients undergoing liver transplantation evaluations. Outside of this context, clinicians recommended that patients seek care for OUD and TUD with primary care providers or addiction specialists. Overall, hepatology clinics were felt to be an important context in which patients may be more open to receiving addiction treatment.

Many clinicians described logistical concerns, such as limited time during clinic visits and competing priorities in patients with complications of liver disease, as barriers to incorporating addiction treatment into their workflow. Fellows felt that consistency in training and comfort among hepatology attendings in addressing addiction among patients with liver disease would help support their learning and ability to incorporate this dimension into patient encounters. Several clinicians raised system-level barriers such as a lack of dedicated professionals to provide behavioral treatments, issues related to reimbursement of addiction care, and challenges in referring patients to addiction care, given shortages of addiction specialists and wait times. Clinicians also felt patient perspectives related to addiction treatment delivery were unclear and should be considered, and that social stressors may affect patients’ ability to participate or benefit from addiction treatment in the absence of more comprehensive psychosocial support. Finally, the role of stigma at the patient-, clinician-, and system-level as a barrier was emphasized.

#### Themes within the facilitation domain

Several themes highlighted opportunities to improve addiction treatment delivery in hepatology clinics. There was considerable variability among clinicians regarding what was felt to be the most feasible and effective model of integrated addiction and hepatology care. Some felt that addiction treatment was beyond the scope of hepatology practice and that dedicated addiction specialists should be incorporated into clinics such that patients can be easily referred. Others felt that changes in professional culture and training could lead to AUD treatment being incorporated into routine hepatology care and that directly providing MAUD alongside referrals for behavioral treatments would be a preferred model of care and more acceptable to patients. Most felt that a multidisciplinary approach, including dedicated behavioral health and addiction specialists, would be ideal, and that system-level investments in resources such as additional personnel and standardized addiction treatment procedures within hepatology clinics would be needed. Some felt that resources such as clinical decision support tools could help guide the provision of MAUD and that tailored education through didactic lectures and longitudinal mentorship by addiction specialists would be valuable. Overall, themes within this domain highlighted the need for flexible models of care that can adapt to and support individual clinicians who may have varying degrees of comfort in providing addiction care.

## DISCUSSION

To our knowledge, this is the first study to assess hepatology clinician perspectives on integrated care for AUD, OUD, and TUD in hepatology clinics using a comprehensive mixed-methods approach. We found that while most hepatology clinicians providing care for patients with chronic liver disease across multiple sites affiliated with an academic center felt that it was important to provide integrated addiction treatment, treatment of AUD was prioritized over treatment for OUD and TUD. In addition, there was greater readiness to prescribe MAUD and less readiness to prescribe MOUD among clinicians with prescribing abilities. Finally, although most clinicians preferred that a dedicated addiction treatment specialist be embedded within hepatology clinics to provide integrated addiction treatment, half felt that hepatology clinicians themselves should directly provide AUD treatment. Perspectives regarding the optimal strategies for delivering addiction treatment in hepatology clinics and for increasing clinician readiness varied greatly, highlighting the need for flexible and adaptable approaches tailored to individual clinical settings.

Previous studies indicate that the majority of clinicians do not prescribe MAUD in part due to low levels of knowledge and comfort, and in one survey-based study, 90% felt that additional training on treating AUD among hepatology clinicians was needed.^21^^,^^41^ Our findings are consistent with prior literature in that similar barriers and facilitators to providing addiction treatment were identified.^24^^,^^42^ Aside from limited knowledge and concerns regarding the safety of MAUD, previously described factors such as time constraints, limited resources, patient complexity, stigma, and the perception of addiction treatment as being beyond the scope of hepatology care also emerged as barriers in our study, and appreciation for the importance of AUD treatment for liver-related outcomes was identified as a facilitator.^24^ By examining clinician perspectives through the lens of the PARiHS framework and by focusing on OUD and TUD in addition to AUD, our study builds upon the existing literature^23^^,^^43^ and additionally identifies evidence and context-related barriers such as the desire for more research to guide addiction treatment for patients with advanced liver disease, limited knowledge related to motivational interviewing and harm reduction in addition to medications, the need for hepatology attending guidance to support trainee experiences in providing addiction treatment, the relative lack of emphasis on OUD and TUD despite their impact on liver health, and structural challenges related to limited time and competing priorities that affect the ability of clinicians to also address addiction in greater detail.

Interventions to support hepatologist-driven models of integrated addiction treatment, particularly for AUD, are emerging. A recent educational intervention aimed at improving hepatology clinician knowledge, attitudes, and practices toward screening and treatment of AUD led to significant improvements in comfort, but not in intentions to prescribe MAUD.^44^ Approaches informed by implementation science that provide tailored multifaceted support to promote evidence-based practices are promising, and various strategies have been used in primary care, emergency department, and subspecialty HIV clinical settings to increase addiction treatment.^32^^,^^33^^,^^45^^–^^49^ A recent multicenter study using implementation facilitation to support the provision of medications for addiction treatment in HIV care settings led to improvements in MAUD and MTUD.^28^ Our findings identified facilitation opportunities to promote uptake of addiction treatment in hepatology clinics. Specifically, the variability in perspectives on models of integrated care that may be most feasible and effective, as well as the emphasis on the need for multidisciplinary teams, clinical decision support tools, better understanding of community resources, and diverse methods of supporting clinicians through mentorship and training opportunities, should be taken into account when designing interventions to incorporate addiction treatment into hepatology clinics.

This study has several strengths. By using both quantitative and qualitative methodologies in which qualitative findings complemented and expanded upon trends seen in the quantitative data, this study provides a detailed, nuanced understanding of clinicians’ experiences, attitudes, readiness, and preferences toward integrated care for addiction and chronic liver disease. In addition, study participants consisted of an interprofessional group of clinicians, including hepatology attendings, fellows, nurses, advanced practice providers, and behavioral health specialists, all of whom were actively involved in the care of patients with liver disease and shared unique perspectives. Furthermore, there was a high level of engagement from participants, with a 94% response rate to the survey and with 35 out of 50 clinicians, including stakeholders and representatives from all professions, participating in interviews. Our study was grounded within PARiHS to facilitate the translation of findings from this formative evaluation to the design of implementation facilitation interventions.

Several limitations should also be considered. All clinicians in this study were affiliated with a single academic center, potentially limiting the generalizability of our findings. Although the response rate and level of engagement in the study were high, the overall sample size was limited, and the quantitative segment of the study was, therefore, not powered to assess for associations between clinician characteristics and outcomes such as practice patterns, attitudes, readiness and preferences toward integrated care for addiction and chronic liver disease. While a substantial addiction treatment gap remains across all substance use disorders,^50^ improvements in rates of MAUD uptake among patients with liver disease may have occurred since the completion of this study.^11^^,^^25^ Finally, we did not explore the perspectives of patients in this study, and although prior studies have focused on this important question,^22^^,^^24^ additional research is needed to further characterize patient preferences regarding integrated care for addiction and chronic liver disease.

## CONCLUSIONS

Addiction can lead to and exacerbate chronic liver diseases, and addiction treatment is associated with liver-related health benefits. We found that most hepatology clinicians participating in this mixed-methods formative evaluation feel it is important for patients with chronic liver disease to receive addiction treatment and feel more ready to treat AUD compared with OUD and TUD. Most favored incorporation of addiction specialists into hepatology clinics, with many also preferring that hepatology clinicians play a direct role in delivering care for AUD in particular. Time constraints, competing priorities, resource allocation, and the need for clinical tools and additional support will need to be considered when designing interventions to promote integrated care for patients with addiction and chronic liver disease. Research focusing on strategies such as multifaceted integration or implementation facilitation that can be tailored to individual clinical settings is needed.

## Supplementary Material
