# Supplementary material for: Hepatology clinician readiness to provide addiction treatment in hepatology clinics: A mixed-methods formative evaluation
Source: Hepatol Commun. 2025 Oct 14;9(11):e0818. doi: 10.1097/HC9.0000000000000818 (PMC12520222; doi:10.1097/HC9.0000000000000818)
Supplement: Supplementary file 1 [file hc9-9-e0818-s001.docx]

**Survey Instrument**

*Thank you for participating in this survey on the perspectives of hepatology providers on addiction treatment for patients with substance use disorders and liver disease. Participation in this survey is voluntary. All responses will be confidential. Please answer all questions to the best of your knowledge.*

1. How many years since your terminal degree have you been caring for patients with liver disease? ___
2. How many patients with liver disease do you care for in a typical week at your outpatient clinic?
3. Among your patients with liver disease in your outpatient clinic, what percentage have the following conditions?

|  | <25% | 25-49% | 50-75% | >75% |
| --- | --- | --- | --- | --- |
| Alcohol-Associated Liver Disease |  |  |  |  |
| Hepatitis C Infection |  |  |  |  |
| Alcohol Use Disorder |  |  |  |  |
| Opioid Use Disorder |  |  |  |  |
| Tobacco Use Disorder |  |  |  |  |

1. Among your patients with liver disease in your outpatient clinic, how often, or what percentage of time, do you ask about the following?

|  | <25% | 25-49% | 50-75% | >75% |
| --- | --- | --- | --- | --- |
| Alcohol Use |  |  |  |  |
| Opioid Use |  |  |  |  |
| Tobacco Use |  |  |  |  |

1. What percentage of your patients with liver disease and the following disorders do you refer for further assessment of substance use or addiction treatment?

|  | <25% | 25-49% | 50-75% | >75% |
| --- | --- | --- | --- | --- |
| Alcohol Use Disorder |  |  |  |  |
| Opioid Use Disorder |  |  |  |  |
| Tobacco Use Disorder |  |  |  |  |

1. Where are patients with liver disease and alcohol, opioid, or tobacco use disorders in your outpatient clinic referred for further assessment of substance use or addiction treatment (check off all that apply)?

__ Social worker within your clinic

__ Psychology or psychiatry services within your clinic

__ Addiction specialist within your health care system

__ Psychology or psychiatry services within the community

__ Addiction treatment program within the community

__ Other (Please Specify: ______________)

__ Patients are not referred for substance use or addiction treatment.

1. [For prescribers of medications such as attendings, fellows, and advance practice providers.] Which of the following medications have you ever prescribed for patients with liver disease and alcohol use disorder for the treatment of addiction (check off all that apply)?

__ Disulfuram

__ Oral naltrexone (Revia)

__ Injectable naltrexone (Vivitrol)

__ Acamprosate (Campral)

__ Baclofen (Lioresal)

__ Topiramate (Topamax)

__ Gabapentin (Neurontin)

__ None

1. [For prescribers of medications such as attendings, fellows, and advance practice providers.] Which of the following medications have you ever prescribed for patients with liver disease and opioid use disorder for the treatment of addiction (check off all that apply)?

__ Buprenorphine (Suboxone)

__ Injectable naltrexone (Vivitrol)

1. [For prescribers of medications such as attendings, fellows, and advance practice providers.] Which of the following medications have you ever prescribed for patients with liver disease and tobacco use disorder for the treatment of addiction (check off all that apply)?

__ Nicotine Patch (Nicoderm, Nicotrol)

__ Nicotine Gum or Lozenge (Nicorette)

__ Nicotine Inhaler (Nicotrol)

__ Varenicline (Chantix)

__ Bupropion (Wellbutrin)

__ None

1. How effective do you think medication treatment is for patients with liver disease for the following disorders? (1 = not effective at all, 5 = very effective)

|  | 1 | 2 | 3 | 4 | 5 |
| --- | --- | --- | --- | --- | --- |
| Alcohol Use Disorder |  |  |  |  |  |
| Opioid Use Disorder |  |  |  |  |  |
| Tobacco Use Disorder |  |  |  |  |  |

1. How important do you think it is to provide treatment for the following conditions for patients with liver disease within hepatology clinics? (1 = not at all important, 5 = very important)

|  | 1 | 2 | 3 | 4 | 5 |
| --- | --- | --- | --- | --- | --- |
| Alcohol Use Disorder |  |  |  |  |  |
| Opioid Use Disorder |  |  |  |  |  |
| Tobacco Use Disorder |  |  |  |  |  |

1. [For prescribers of medications such as attendings, fellows, and advance practice providers.] How ready do you feel to provide medication treatment for the following conditions for your patients with liver disease? (1 = not ready, 5 = very ready)

|  | 1 | 2 | 3 | 4 | 5 |
| --- | --- | --- | --- | --- | --- |
| Alcohol Use Disorder |  |  |  |  |  |
| Opioid Use Disorder |  |  |  |  |  |
| Tobacco Use Disorder |  |  |  |  |  |

1. What would be your preferred model of care for providing treatment for alcohol use disorder within hepatology clinics?

__ All liver clinicians in the practice provide treatment for alcohol use disorder for the patients in their panel

__ One or more liver clinicians in the practice are designated as the specialists and receive referrals for alcohol use disorder from other providers in the practice

__ An outside addiction specialist is incorporated into the clinic to provide alcohol use disorder treatment for all eligible patients

__Patients are referred to addiction treatment for alcohol use disorder outside the practice

1. What would be your preferred model of care for providing treatment for opioid use disorder within hepatology clinics?

__ All liver clinicians in the practice provide treatment for opioid use disorder for the patients in their panel

__ One or more liver clinicians in the practice are designated as the specialists and receive referrals for opioid use disorder from other providers in the practice

__ An outside addiction specialist is incorporated into the clinic to provide opioid use disorder treatment for all eligible patients

__Patients are referred to addiction treatment for opioid use disorder outside the practice

1. What would be your preferred model of care for providing treatment for tobacco use disorder within hepatology clinics?

__ All liver clinicians in the practice provide treatment for tobacco use disorder for the patients in their panel

__ One or more liver clinicians in the practice are designated as the specialists receive referrals for tobacco use disorder from other providers in the practice

__ An outside addiction specialist is incorporated into the clinic to provide tobacco use disorder treatment for all eligible patients

__Patients are referred to addiction treatment for tobacco use disorder outside the practice

1. What type of clinician are you?

__Advanced Practice Provider

__Attending Hepatologist

__Attending Psychiatrist

__Fellow

__Nurse

__Social Worker

__Psychologist

1. What is your primary clinical focus?

__Alcohol-Associated Liver Disease

__Autoimmune and Cholestatic Liver Diseases

__Cirrhosis and Portal Hypertension

__Drug Induced Liver Injury

__Fatty Liver Disease

__Liver Cancer

__Liver Transplant

__Metabolic Liver Diseases

__Viral Hepatitis

__Other (Please Specify: ______________)

1. What is your gender?

__Woman

__Man

__Transgender

__Non-Binary

__Prefer Not to Say

1. What year were you born? ____
2. What year did you complete your terminal degree? ____
3. With which racial groups do you identify?

__Native American or Alaskan Native

__Native Hawaiian or Other Pacific Islander

__Asian

__Black or African American

__White

__Other (Please Specify: ______________)

__Prefer Not to Say

1. With which ethnic groups do you identify?

__Hispanic or Latinx

__Not Hispanic or Latinx

__Prefer Not to Say

1. Which of the following team members are included in your practice setting (check off all that apply)?

__Social workers

__Nurse coordinator

__Psychologists or psychiatrists

__Clinical pharmacists

__Other (Please Specify: ______________)

__None

24. What type of addiction medicine training have you previously received?

__Didactic Lecture

__Outpatient Clinical Rotation

__Inpatient Clinical Rotation

__Continuing Medical Education Activity

__Other

__None

**Qualitative Interview Guide**

*Introduction: This study is intended to understand your perspective regarding factors that influence the delivery of treatment for alcohol, tobacco, and opioid use disorders through your hepatology clinic. The goal of this study is to use your input to inform the development of interventions to improve how such care can be provided through the hepatology clinic. Everything that is said here will be kept confidential and I will ask that others do the same. We will only share a summary of these findings with others with the goal of improving the experiences of providers and patients with regards to the delivery of addiction treatment in this setting.*

1. To get started, can you tell me generally about your understanding of how much of a need there is for treatment of alcohol use disorders in your clinic? What about tobacco use disorders? And opioid use disorders?

*Prompts*

- 1. *To what extent do you think these addictions impact your patients?*
  2. *How comfortable are you in assessing your patients for the presence of these disorders?*
  3. *What has been your experience addressing these disorders in your patients?*

1. Can you tell me, what is your understanding of the evidence for treating alcohol use disorder among patients with liver disease? What about tobacco use disorder? Opioid use disorder?

*Prompts*

- 1. *What type of evidence comes to mind? (clinical trials, observational studies, guidelines)*
  2. *How relevant do you think this evidence is for your patients? What makes it more or less relevant for your patients?*
  3. *What motivates you to apply this evidence? What makes it hard to apply this evidence?*
  4. *What type of support do you have to apply this evidence?*

1. What about patients – what do you think patients know about this evidence? What do you think would help them apply this evidence to themselves?

*Prompts*

- 1. *How relevant do you think your patients see this evidence as applicable to them?*
  2. *What do you think impacts how patients apply this evidence to themselves?*

1. What about your clinic directors, administrators, and those who pay for the care your patients receive – to what extent do you think they are aware of this evidence for treatment of alcohol use disorder? How about tobacco use disorder? Opioid use disorder?

*Prompts*

- 1. *How relevant do you think your clinical directors and administrators see this as an important issue?*
  2. *What do you think impacts how they prioritize this evidence?*
  3. *To what extent do you think treatment of tobacco, alcohol, or opioid use disorders is measured as a performance status?*

1. How do you think adopting evidence-based practices for the treatment of alcohol use disorder would impact your clinical practice? What about outcomes for your patients? And what about for tobacco use disorder? Opioid use disorder?

*Prompts*

- 1. *How ready or comfortable do you feel to incorporate this practice?*
  2. *What would make it hard to do this? What would make it easier to do this?*
  3. *How important do you think it is to do this in your clinic?*
  4. *What training would you need to do this?*
  5. *What systems would you need in place to do this?*
  6. *What types of staff changes would you need in your clinic to do this?*
  7. *What types of performance measures are currently in place? What kind of feedback do you get about this to inform your practice?*
  8. *How would this affect the care of patients in the context of liver transplantation?*

1. What types of resources do you currently have for addressing alcohol use disorder in your clinic? Tobacco use disorder? Opioid use disorder?

*Prompts*

- 1. *What is your present system, if any, of addressing these disorders?*
  2. *What would be the ideal system?*
  3. *What would you need to make that happen?*
  4. *How much support is there for such a change?*
